# Supplementary material for: Modulation of autoimmune pathogenesis by T cell-triggered inflammatory cell death
Source: Nat Commun. 2019 Aug 28;10:3878. doi: 10.1038/s41467-019-11858-7 (PMC6713751; doi:10.1038/s41467-019-11858-7)
Supplement: Supplementary file 1 — Supplementary Information [file 41467_2019_11858_MOESM1_ESM.pdf]

Supplementary Information

Modulation of autoimmune pathogenesis by T cell-triggered inflammatory cell death

Sasaki et al.

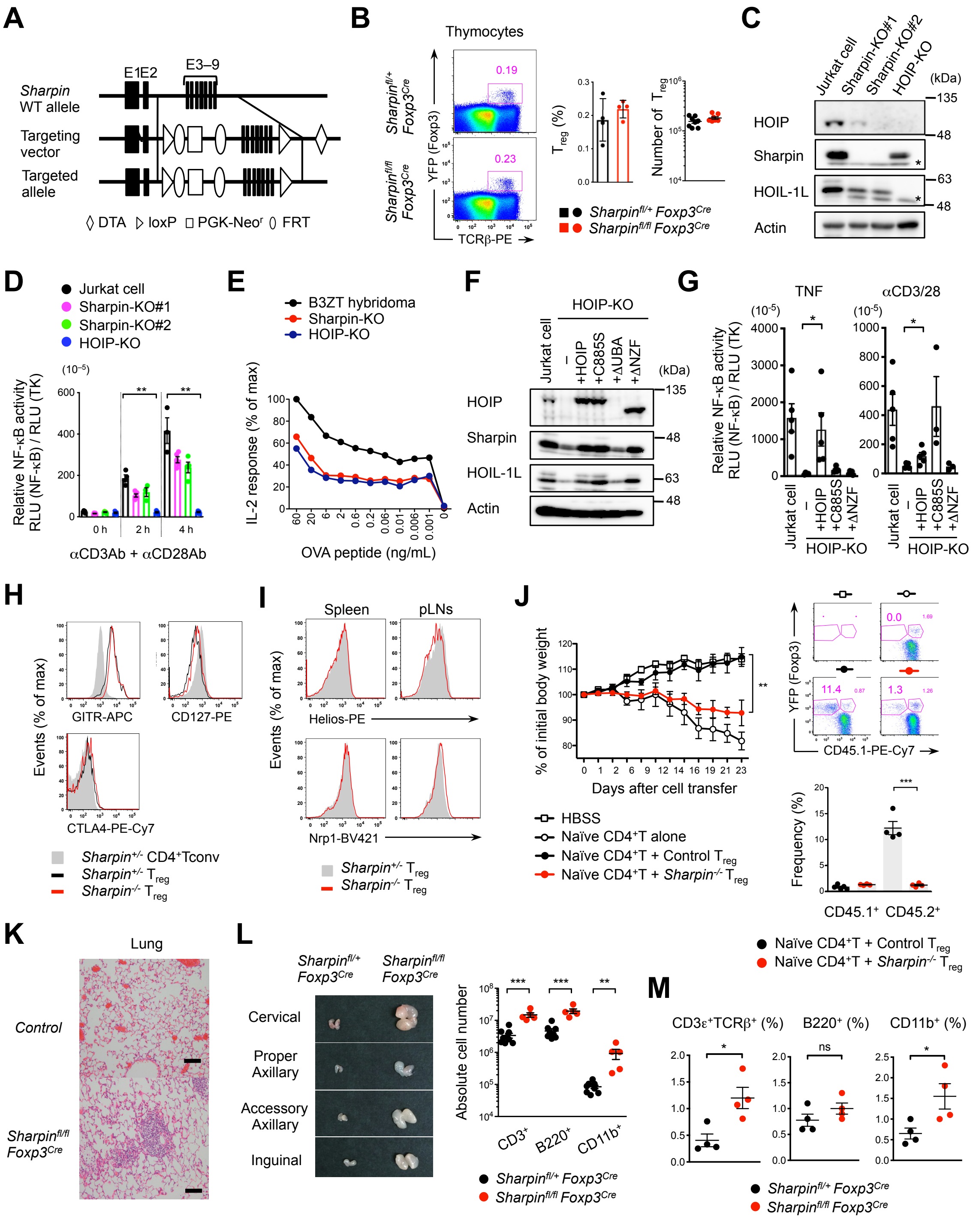

A
Sharpin WT allele
Targeting vector
Targeted allele
E1E2
E3–9
◊ DTA ▷ loxP □ PGK-Neor () FRT
B
Thymocytes
Sharpinfl/+ Foxp3Cre
Sharpinfl/fl Foxp3Cre
YFP (Foxp3)
TCRβ-PE
0.19
0.23
Treg (%)
Number of Treg
C
Jurkat cell
Sharpin-KO#1
Sharpin-KO#2
HOIP-KO
(kDa)
HOIP
Sharpin
HOIL-1L
Actin
D
Jurkat cell
Sharpin-KO#1
Sharpin-KO#2
HOIP-KO
Relative NF-κB activity RLU (NF-κB) / RLU (TK)
0 h
2 h
4 h
αCD3Ab + αCD28Ab
E
B3ZT hybridoma
Sharpin-KO
HOIP-KO
IL-2 response (% of max)
OVA peptide (ng/mL)
F
HOIP-KO
Jurkat cell
+HOIP
+C885S
+ΔUBA
+ΔNZF
HOIP
Sharpin
HOIL-1L
Actin
G
TNF
αCD3/28
Relative NF-κB activity RLU (NF-κB) / RLU (TK)
HOIP-KO
H
GITR-APC
CD127-PE
CTLA4-PE-Cy7
Events (% of max)
Sharpin+/- CD4+Tconv
Sharpin+/- Treg
Sharpin-/- Treg
I
Spleen
pLNs
Helios-PE
Nrp1-BV421
Events (% of max)
Sharpin+/- Treg
Sharpin-/- Treg
J
% of initial body weight
Days after cell transfer
HBSS
Naïve CD4+T alone
Naïve CD4+T + Control Treg
Naïve CD4+T + Sharpin-/- Treg
YFP (Foxp3)
CD45.1-PE-Cy7
Frequency (%)
CD45.1+ CD45.2+
K
Lung
Control
Sharpinfl/fl Foxp3Cre
L
Sharpinfl/+ Foxp3Cre
Sharpinfl/fl Foxp3Cre
Cervical
Proper Axillary
Accessory Axillary
Inguinal
Absolute cell number
CD3+
B220+
CD11b+
M
Naïve CD4+T + Control Treg
Naïve CD4+T + Sharpin-/- Treg
CD3ε+TCRβ+ (%)
B220+ (%)
CD11b+ (%)
Sharpinfl/+ Foxp3Cre
Sharpinfl/fl Foxp3Cre

Supplementary Fig. 1. LUBAC dependency during TCR-mediated NF-κB signaling, and the phenotypes of *Sharpin*$^{fl/fl}$*Foxp3*^{Cre}.

(A) Structure of *Sharpin* genomic wild-type alleles, along with the targeting vectors and targeted alleles. Solid boxes, open boxes, open triangles, open rhombuses, and open ovals denote the coding exons, the neomycin resistance gene, loxP sites, diphtheria toxin A, and FRT sites, respectively. (B) Absolute cell number (n = 4) and percentage (n = 8) of Foxp3^{+} thymocytes in 4-week-old *Sharpin*$^{fl/fl}$*Foxp3*^{Cre} and control mice. (C) Immunoblot analysis of Sharpin- or HOIP-deficient Jurkat cells. Asterisks indicate non-specific bands. (D) TCR-mediated NF-κB signaling in response to CD3/28 antibodies. (E) IL-2 secretion was estimated using IL-2-dependent HT-2 cells. A B3Z T cell hybridoma, which expresses an OVA peptide-K^{b}-specific pair of TCRαβ chains, and the corresponding Sharpin- or HOIP-deficient cells were stimulated with cognate antigens at various concentrations in the presence of MHC I-expressing cells. (F) Immunoblot analysis of HOIP mutant-expressing HOIP-KO Jurkat cells. Asterisks indicate non-specific bands. (G) Intensity of NF-κB signaling was measured after stimulation by TNF or CD3/28 antibodies. (H) Representative histogram showing expression of GITR, CD127, and CTLA4 on the surface of Foxp3^{-}CD4^{+} T and T_{reg}. (I) Representative histogram showing expression of Nrp1 and intracellular Helios by T_{reg} from the spleen and peripheral LNs (pLNs) of 10-week-old *Sharpin*$^{fl/fl}$*Foxp3*^{Cre} and control mice. (J) T_{reg}-mediated suppression assay using sorted naïve (CD45.1^{+}CD25^{-}CD45RB^{hi}) CD4^{+} T cells and T_{reg} (CD45.2^{+}YFP^{+}CD25^{+}CD45RB^{lo}). Colitis-dependent weight loss in *Rag2*$^{-/-}$ mice given no T cells (open squares), naïve T cells alone (black circles), or a combination of naïve T cells and WT T_{reg} (open circles) or *Sharpin*$^{-/-}$ T_{reg} (red circles). Data are pooled from five to six mice per group. Representative plots show transferred CD45.1^{-}CD45.2^{+} T_{reg} and the percentage of T_{reg} among the CD4^{+} T cell population within the mesenteric lymph nodes. n = 5 biologically independent animals per each group. (K) Immune cell infiltration of the lung. Scale bar: 200 μm. (L) Representative photos of enlarged pLNs (left) and absolute number of the indicated immune cells in pLNs (right) from 10-week-old *Sharpin*$^{fl/fl}$*Foxp3*^{Cre} and control mice. n = 5 biologically independent animals. (M) Percentage of the indicated epidermal infiltrates in the skin of 4-week-old *Sharpin*$^{fl/fl}$*Foxp3*^{Cre}. n = 4 biologically independent animals. Small

circles in the graphs indicate data from an individual mouse. Small horizontal lines indicate the mean (± s.e.m.). *$p < 0.05$, **$p < 0.01$, ***$p < 0.001$. Kruskal-Wallis test with Bonferroni correction (α value = 0.05) was used for D, G, and J (left), while two-tailed Mann-Whitney U-test was used for J (right), L, and M. Data are pooled from at least three independent experiments (B, J, L, and M). Source data are provided in a Source Data file.

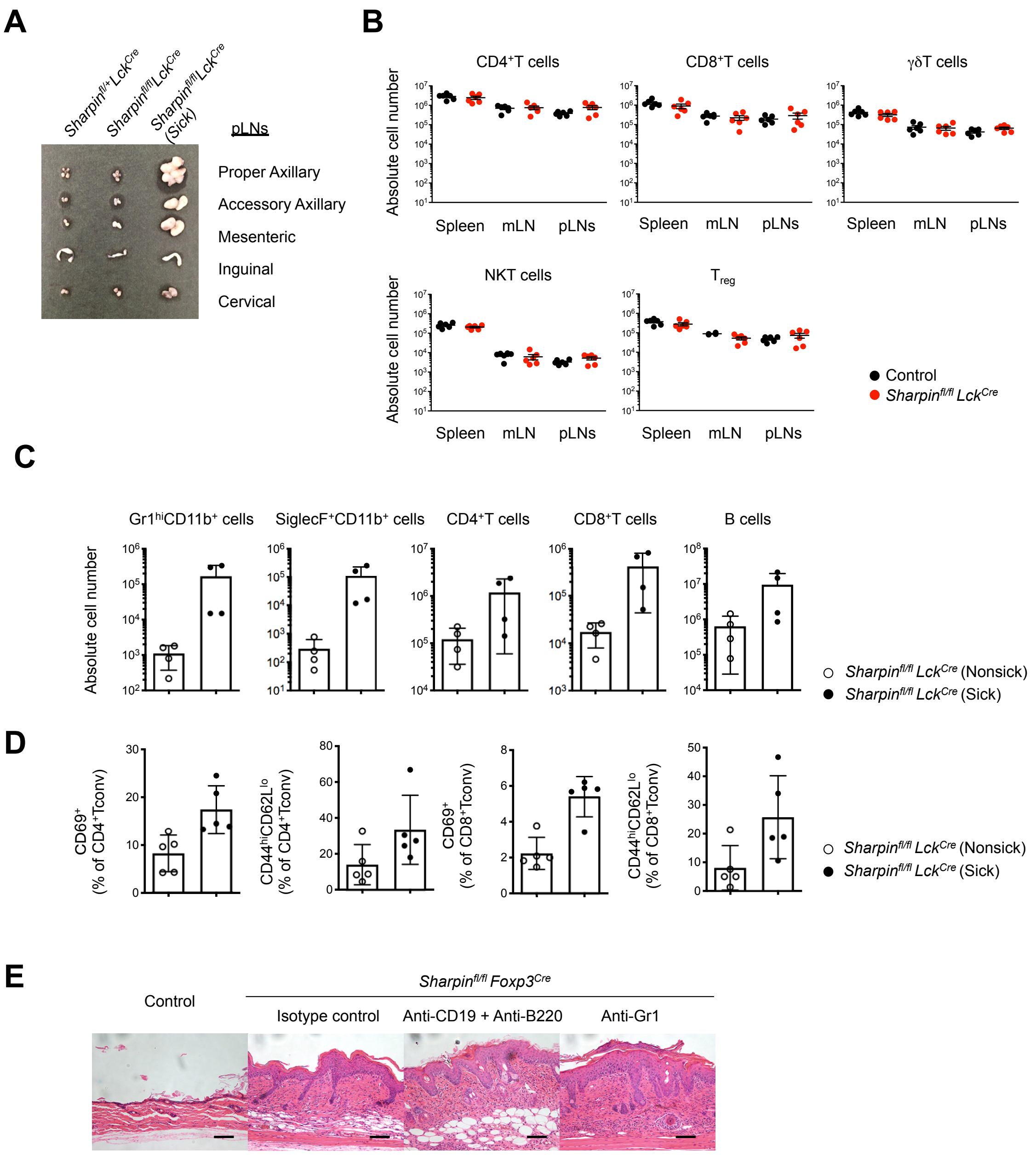

Supplementary Fig. 2. Phenotypes of *Sharpin*$^{fl/fl}$*Lck*^{Cre}.

(A) Representative photos of pLNs. (B) Absolute numbers of the indicated T cell subsets in the spleen, mLN, and pLNs of healthy *Sharpin*$^{fl/fl}$*Lck*^{Cre} and control mice. (C) Absolute number of the indicated immune cells in pLNs from nonsick and Sick *Sharpin*$^{fl/fl}$*Lck*^{Cre}

mice. (D) Percentage of $CD69^+$ or $CD44^{hi}CD62L^{lo}$ activated conventional T cells in pLNs (upper: $CD4^+$T cells; lower: $CD8^+$T cells). (E) Administration of antibodies to achieve B or myeloid cell depletion from *Sharpin*$^{fl/fl}$*Foxp3*^{Cre} mice. Scale bar: 200 μm. Small horizontal lines indicate the mean (± s.e.m.). Source data are provided in a Source Data file.

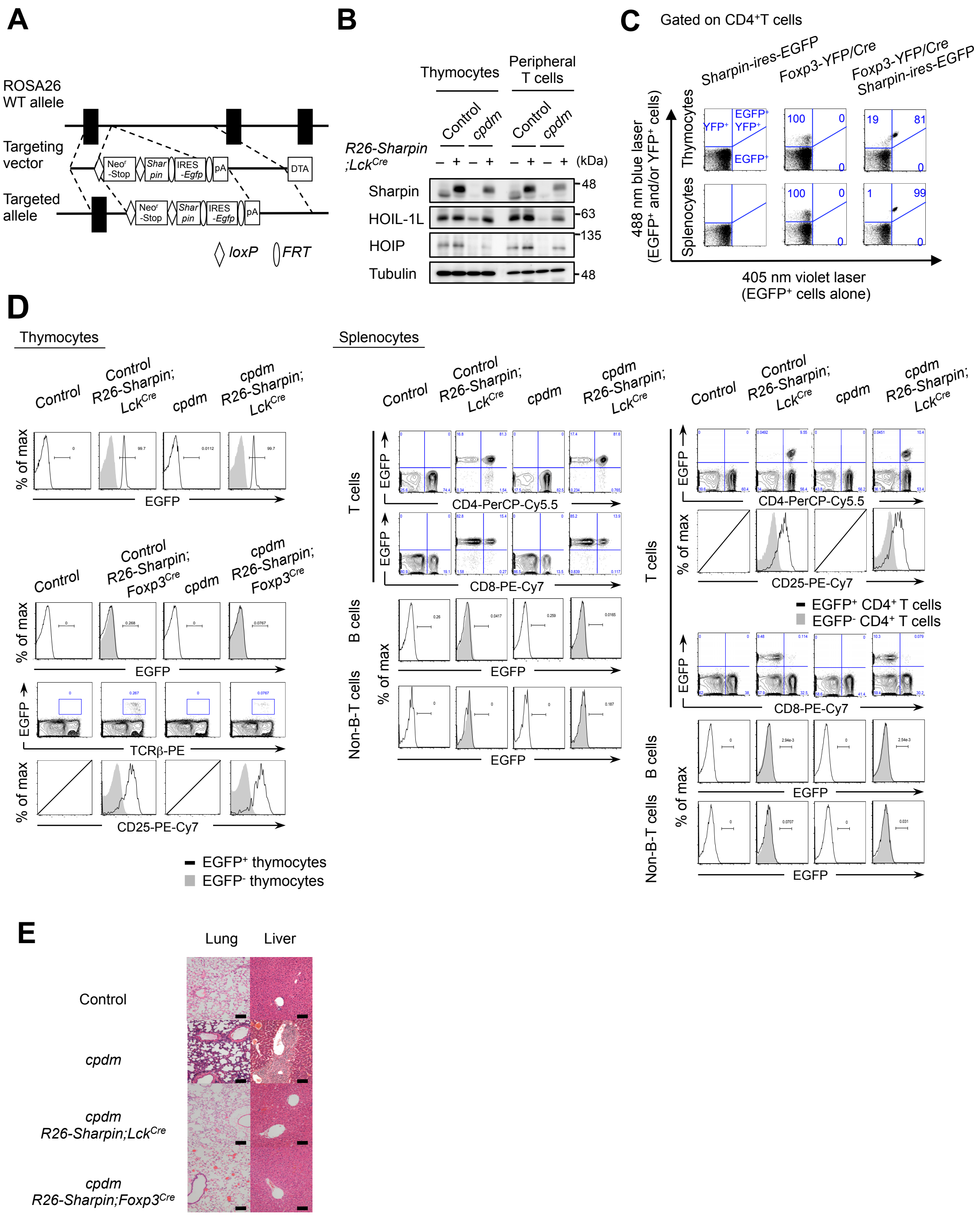
A
ROSA26 WT allele
Targeting vector
Targeted allele
Neo^r -Stop
Sharpin
IRES -Egfp
pA
DTA
loxP
FRT
B
Thymocytes
Peripheral T cells
Control
cpdm
R26-Sharpin ;Lck^Cre
– + – +
(kDa)
Sharpin
HOIL-1L
HOIP
Tubulin
48
63
135
48
C
Gated on CD4+T cells
Sharpin-ires-EGFP
Foxp3-YFP/Cre
Foxp3-YFP/Cre Sharpin-ires-EGFP
Thymocytes
Splenocytes
YFP+
EGFP+ YFP+
EGFP+
100
0
19
81
1
99
488 nm blue laser (EGFP+ and/or YFP+ cells)
405 nm violet laser (EGFP+ cells alone)
D
Thymocytes
Splenocytes
Control
Control R26-Sharpin; Lck^Cre
cpdm
cpdm R26-Sharpin; Lck^Cre
Control R26-Sharpin; Foxp3^Cre
cpdm R26-Sharpin; Foxp3^Cre
% of max
EGFP
TCRβ-PE
CD25-PE-Cy7
CD4-PerCP-Cy5.5
CD8-PE-Cy7
T cells
B cells
Non-B-T cells
EGFP+ thymocytes
EGFP- thymocytes
EGFP+ CD4+ T cells
EGFP- CD4+ T cells
E
Lung
Liver
Control
cpdm
cpdm R26-Sharpin;Lck^Cre
cpdm R26-Sharpin;Foxp3^Cre

Supplementary Fig. 3. Validation of Sharpin conditional knock-in mice.

(A) Schematic showing the gene-targeting strategy used to generate conditional *Rosa26-Sharpin-ires-Egfp* transgenic mice. (B) Immunoblot analyses to detect LUBAC components in thymocytes or peripheral T cells from 4-week-old *cpdm* and control mice with or without expression of T cell-specific Sharpin (*R26-Sharpin;Lck*^{Cre}). Bands representing genetically transduced Sharpin proteins have a higher molecular weight than endogenous proteins due to tagging with FLAG-His_6. Tubulin was used as a loading control. (C) Validation of Cre recombinase-mediated expression by *Sharpin-ires-Egfp* in *R26-Sharpin;Foxp3*^{Cre} mice. Thymocytes and splenocytes from 4-week-old mice harboring the indicated alleles were analyzed by flow cytometry. The 488 nm blue laser excites both EGFP and YFP, whereas the 405 nm violet laser excites EGFP, but not YFP. Accordingly, $EGFP^+$/YFP^+ or YFP^+ cells denote Foxp3 promoter-driven Cre recombinase-expressing cells with or without the *Sharpin* transgene alleles, respectively. The numbers inside the gated areas indicate the percentage of cells excited by the 488 nm blue laser. (D) Flow cytometry analysis of $EGFP^+$ cells in the thymocyte and splenocyte populations from 4-week-old mice. Expression of the Sharpin transgene was estimated from the intensity of the reporter EGFP signal. Detected $EGFP^+CD4^+$ T cells in control *R26-Sharpin;Foxp3*^{Cre} and cpdm *R26-Sharpin;Foxp3*^{Cre} mice were identified predominantly as $CD25^+$ T_{reg}. Representative data are derived from three independent experiments, each with similar results. (E) Representative sections of lung and liver from 12-week-old mice stained with H/E. Scale bars: 100 μm. Data are derived from three independent experiments. Source data are provided in a Source Data file.

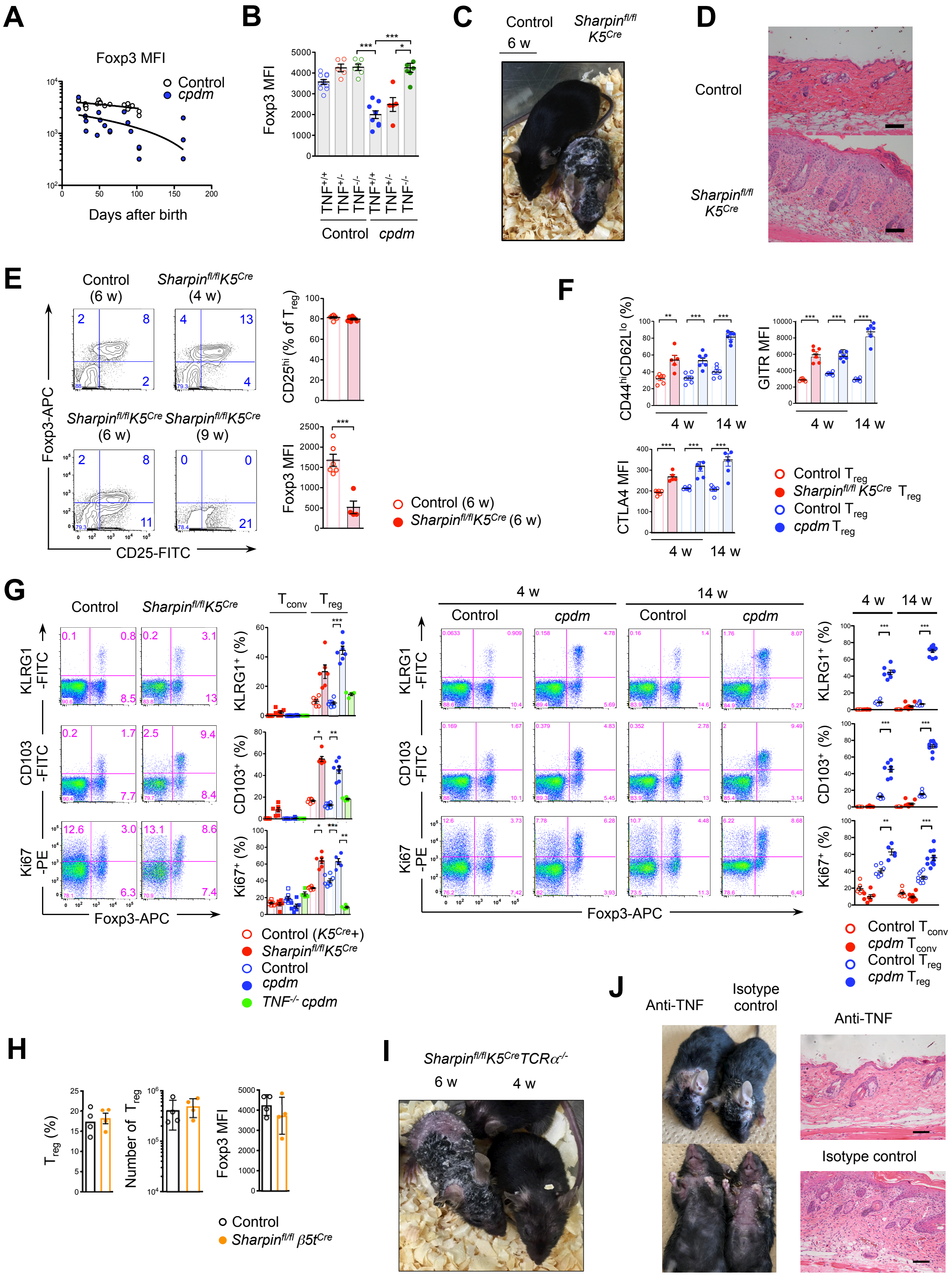
A
Foxp3 MFI
Control
cpdm
Days after birth
B
Foxp3 MFI
TNF+/+ TNF+/- TNF-/- TNF+/+ TNF+/- TNF-/-
Control cpdm
C
Control 6 w
Sharpin fl/fl K5Cre
D
Control
Sharpin fl/fl K5Cre
E
Control (6 w)
Sharpin fl/fl K5Cre (4 w)
Sharpin fl/fl K5Cre (6 w)
Sharpin fl/fl K5Cre (9 w)
Foxp3-APC
CD25-FITC
CD25hi (% of Treg)
Foxp3 MFI
Control (6 w)
Sharpin fl/fl K5Cre (6 w)
F
CD44hiCD62Llo (%)
GITR MFI
CTLA4 MFI
4 w 14 w
Control Treg
Sharpin fl/fl K5Cre Treg
Control Treg
cpdm Treg
G
Control
Sharpin fl/fl K5Cre
KLRG1-FITC
CD103-FITC
Ki67-PE
Foxp3-APC
Tconv Treg
KLRG1+ (%)
CD103+ (%)
Ki67+ (%)
Control (K5Cre+)
Sharpin fl/fl K5Cre
Control
cpdm
TNF-/- cpdm
4 w 14 w
Control cpdm Control cpdm
Control Tconv
cpdm Tconv
Control Treg
cpdm Treg
H
Treg (%)
Number of Treg
Foxp3 MFI
Control
Sharpin fl/fl β5tCre
I
Sharpin fl/fl K5Cre TCRα-/-
6 w 4 w
J
Anti-TNF
Isotype control

Supplementary Fig. 4. Skin autoinflammation extrinsically induces T_{reg} instability in *Sharpin$^{fl/fl}$K5^{Cre}* and *cpdm* mice.

(A) MFI of Foxp3 in splenic T_{reg} from *cpdm* (n = 21) and control mice (n = 19) aged 3–24 weeks. (B) MFI of Foxp3 in splenic T_{reg} isolated from 4–5-week-old *cpdm* and control mice with a *TNF$^{+/+}$*, *TNF$^{+/-}$*, or *TNF$^{-/-}$* genetic background. (C) Appearance of 6-week-old *Sharpin$^{fl/fl}$K5^{Cre}* and control mice. (D) H/E staining of skin sections. (E) Representative plots showing T_{reg} ($CD4^+CD25^+Foxp3^+$) isolated from the spleen of *Sharpin$^{fl/fl}$K5^{Cre}* and control mice of the indicated age. Percentage of $CD25^{hi}$ T_{reg} (upper right) and MFI of Foxp3 in T_{reg} (lower right) from 6-week-old mice. n = 4 biologically independent animals (F) Percentage of $CD44^{hi}CD62L^{lo}$ (activated phenotype) cells and MFI of GITR or CTLA4 (T_{reg} functional markers) in T_{reg} isolated from 6-week-old *Sharpin$^{fl/fl}$K5^{Cre}* and 4- and 14-week-old *cpdm* mice. n = 6 biologically independent animals. (G) Representative plots (left) and percentages (right) of T_{reg} (isolated from the spleen of 4–5-week-old mice of the indicated strain [left] and 4- and 14-week-old *cpdm* mice [right]) expressing $KLRG1^+$ and $CD103^+$ (activation markers) and $Ki67^+$ (a proliferation marker). n = 7 biologically independent animals. (H) Absolute number and percentage of $Foxp3^+$ T_{reg}, and MFI of Foxp3, in T_{reg} from *Sharpin$^{fl/fl}$β5t^{Cre}* and control mice. n = 4 biologically independent animals. (I) Appearance of *Sharpin$^{fl/fl}$K5^{cre}TCRα$^{-/-}$* mice. (J) Administration of TNF—neutralizing antibody on *Sharpin$^{fl/fl}$K5^{cre}*. Scale bar: 200 μm. Small circles in the graphs indicate data from an individual mouse. Small horizontal lines indicate the mean (± s.e.m.). $**p < 0.01$, $***p < 0.001$. Kruskal-Wallis test with Bonferroni correction (α value = 0.05) was used for B and G, while two-tailed Mann-Whitney U-test was used for E, F and K. Data are pooled from at least three independent experiments. Source data are provided in a Source Data file.

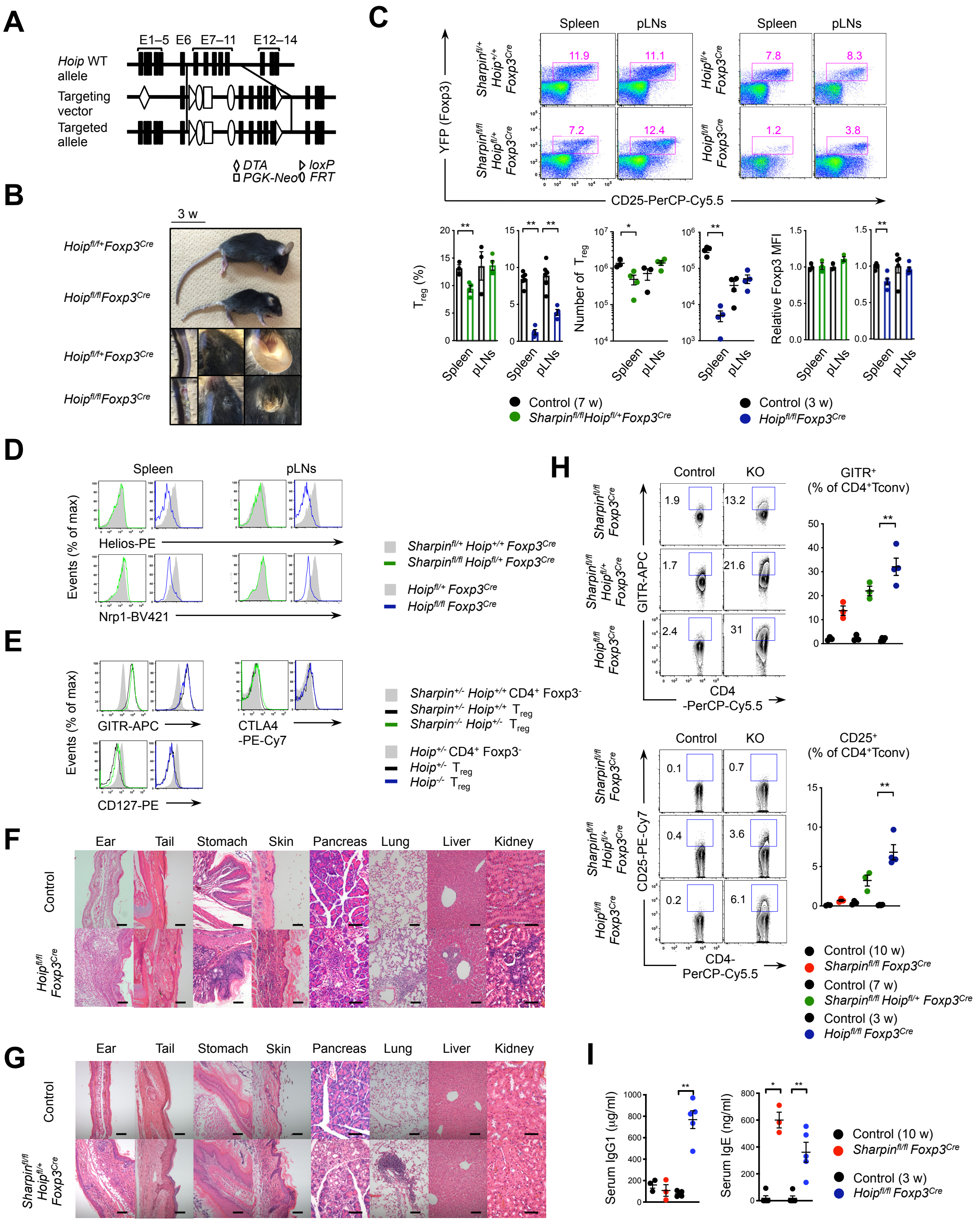
A
Hoip WT allele
Targeting vector
Targeted allele
E1–5 E6 E7–11 E12–14
DTA loxP PGK-Neo FRT
B
3 w
Hoipfl/+Foxp3Cre
Hoipfl/flFoxp3Cre
C
Spleen pLNs
YFP (Foxp3)
CD25-PerCP-Cy5.5
Control (7 w)
Sharpinfl/flHoipfl/+Foxp3Cre
Control (3 w)
Hoipfl/flFoxp3Cre
Number of Treg
Relative Foxp3 MFI
D
Helios-PE
Nrp1-BV421
Events (% of max)
E
GITR-APC
CTLA4 -PE-Cy7
CD127-PE
F
Ear Tail Stomach Skin Pancreas Lung Liver Kidney
Control
G
H
Control KO
GITR-APC
CD4 -PerCP-Cy5.5
GITR+ (% of CD4+Tconv)
CD25-PE-Cy7
CD25+ (% of CD4+Tconv)
Control (10 w)
Sharpinfl/fl Foxp3Cre
I
Serum IgG1 (μg/ml)
Serum IgE (ng/ml)

Supplementary Fig. 5. Phenotypes of different autoimmune murine strains harboring T_{reg} with compromised LUBAC.

(A) Structure of the *Hoip* genomic wild-type alleles, along with the targeting vectors and targeted alleles. Solid boxes, open boxes, open triangles, open rhombuses, and open ovals denote the coding exons, the neomycin resistance gene, loxP sites, diphtheria toxin A, and FRT sites, respectively. (B) Gross appearance of 3-week-old $Hoip^{fl/fl}Foxp3^{Cre}$ and control mice. (C) Percentage of $Foxp3^{+}$ cells within the $CD4^{+}$ T cell population, absolute number of T_{reg}, and the MFI of Foxp3 in T_{reg} in spleen and pLNs from 7-week-old $Sharpin^{fl/fl}Hoip^{fl/+}Foxp3^{Cre}$ and 3-week-old $Hoip^{fl/fl}Foxp3^{Cre}$ mice and their respective controls. $n = 4$ biologically independent animals. (D) Histogram showing expression of Nrp1 and intracellular Helios by T_{reg} from the spleen and pLNs. (E) Histogram showing expression of GITR, CD127, and CTLA4 on the surface of $Foxp3^{-}CD4^{+}$ T and T_{reg} from the indicated strains. (F and G) H/E staining of sections from various organs from 3-week-old $Hoip^{fl/fl}Foxp3^{Cre}$ and control mice (F), or 7-week-old $Sharpin^{fl/fl}Hoip^{fl/+}Foxp3^{Cre}$ and control mice (G). Scale bars: 40 μm for the pancreas and kidney, 100 μm for the others. (H) Representative plots of $GITR^{+}$ or $CD25^{+}$ (activation markers) expression by $Foxp3^{-}CD4^{+}$ T cells isolated from pLNs of 10-week-old $Sharpin^{fl/fl}Foxp3^{Cre}$ and control ($n = 3$), 7-week-old $Sharpin^{fl/fl}Hoip^{fl/+}Foxp3^{Cre}$ and control ($n = 3$), and 3-week-old $Hoip^{fl/fl}Foxp3^{Cre}$ and control mice ($n = 4$). (I) Quantities of serum IgE and IgG1 in 10-week-old $Sharpin^{fl/fl}Foxp3^{Cre}$ and control ($n = 3$), and in 3-week-old $Hoip^{fl/fl}Foxp3^{Cre}$ and control mice ($n = 5$). Small circles in the graphs indicate data from an individual mouse. Small horizontal lines indicate the mean (± s.e.m.). $*p < 0.05$, $**p < 0.01$, $***p < 0.001$. Kruskal-Wallis test with Bonferroni correction (α value = 0.05) was used for H, while two-tailed Mann-Whitney U-test was used for C and I. Data are representative of at least three independent experiments (D–G) or pooled from three independent experiments (C, H, and I). Source data are provided in a Source Data file.
