# Supplementary material for: Modulation of autoimmune pathogenesis by T cell-triggered inflammatory cell death
Source: Nat Commun. 2019 Aug 28;10:3878. doi: 10.1038/s41467-019-11858-7 (PMC6713751; doi:10.1038/s41467-019-11858-7)
Supplement: Supplementary file 3 — Reporting Summary [file 41467_2019_11858_MOESM3_ESM.pdf]

natureresearch

Corresponding author(s): NCOMMS-18-33420A

Last updated by author(s): Jul 26, 2019

Reporting Summary

Nature Research wishes to improve the reproducibility of the work that we publish. This form provides structure for consistency and transparency in reporting. For further information on Nature Research policies, see Authors & Referees and the Editorial Policy Checklist.

Statistics

For all statistical analyses, confirm that the following items are present in the figure legend, table legend, main text, or Methods section.

n/a	Confirmed	
☐	☒	The exact sample size (*n*) for each experimental group/condition, given as a discrete number and unit of measurement
☐	☒	A statement on whether measurements were taken from distinct samples or whether the same sample was measured repeatedly
☐	☒	The statistical test(s) used AND whether they are one- or two-sided<br>*Only common tests should be described solely by name; describe more complex techniques in the Methods section.*
☒	☐	A description of all covariates tested
☐	☒	A description of any assumptions or corrections, such as tests of normality and adjustment for multiple comparisons
☐	☒	A full description of the statistical parameters including central tendency (e.g. means) or other basic estimates (e.g. regression coefficient) AND variation (e.g. standard deviation) or associated estimates of uncertainty (e.g. confidence intervals)
☐	☒	For null hypothesis testing, the test statistic (e.g. *F*, *t*, *r*) with confidence intervals, effect sizes, degrees of freedom and *P* value noted<br>*Give P values as exact values whenever suitable.*
☒	☐	For Bayesian analysis, information on the choice of priors and Markov chain Monte Carlo settings
☒	☐	For hierarchical and complex designs, identification of the appropriate level for tests and full reporting of outcomes
☒	☐	Estimates of effect sizes (e.g. Cohen's *d*, Pearson's *r*), indicating how they were calculated

Our web collection on statistics for biologists contains articles on many of the points above.

Software and code

Policy information about availability of computer code

Data collection	Flow cytometry data was collected by using FACS Diva software ver6.0 (Becton Dickinson). On western blot analyses, chemiluminescent images were acquired using Image reader LAS-4000mini ver2.1 (FUJIFILM). Signal intensity on MTT analyses was measured by SoftMax Pro software ver5.4 (Molecular Devices). Cell survival data on iCelligence system were acquired by using RTCA iCelligence Software (ACEA Biosciences). H/E and MT images were collected using cellSens standard Software (Olympus), and Immunofluorescence images were collected using BZ-II viewer application (Keyence) or FV10-ASW ver1.6 (Olympus). Raw microarray data from GEO database (Accession number GSE108793) was collected using Affymetrix GeneChip Command Console Software 4.0 (Affymetrix), and the data was background corrected and normalized using Microarray Suite ver5.0 (MAS5) algorithm in Affymetrix Expression Console Software 1.4 (Affymetrix).
Data analysis	Graphpad Prism software 5.0c was used for statistical analyses of cell number, a ratio of two types of cells, percentage or MFI of cells analyzed by FACS, survival data, disease severity data, gene expresssion data, and MTT assay data. FlowJo software (Tomy Digital Biology, version 9.9.6) was used for all analyses of flow cytometry data. Image Gauge ver4.22 (FUJIFILM) was used for chemiluminescent images from LAS-4000 Image reader. ViiA7 RUO Software ver1.2.3 (Thermo Fisher Scientific) was used for RNA expression analysis. Recording data of cell survival using iCelligence system was analyzed by RTCA Data Analysis Software 1.0 (ACEA Biosciences). Immunofluorescence images were processed using the BZ-II image analysis application (Keyence). Microarray data was analyzed by using GeneSpring GX (Agilent Technologies). a heatmap for each transcript value was plotted using Excel (Microsoft). Figures in the manuscript were arranged and converted into PDF files by using PowerPoint software (Microsoft).

For manuscripts utilizing custom algorithms or software that are central to the research but not yet described in published literature, software must be made available to editors/reviewers. We strongly encourage code deposition in a community repository (e.g. GitHub). See the Nature Research guidelines for submitting code & software for further information.

Data

Policy information about availability of data

All manuscripts must include a data availability statement. This statement should provide the following information, where applicable:

- Accession codes, unique identifiers, or web links for publicly available datasets
- A list of figures that have associated raw data
- A description of any restrictions on data availability

Microarray data that support the findings of this study have been deposited in GEO with the accession codes GSE108793, which are accessible using token "gzatqeosbdibpqn" while they remain in private status. The authors declare that the other data supporting the findings of this study are available within the paper and its supplementary information files.

Field-specific reporting

Please select the one below that is the best fit for your research. If you are not sure, read the appropriate sections before making your selection.

☒ Life sciences ☐ Behavioural & social sciences ☐ Ecological, evolutionary & environmental sciences

For a reference copy of the document with all sections, see nature.com/documents/nr-reporting-summary-flat.pdf

Life sciences study design

All studies must disclose on these points even when the disclosure is negative.

Sample size	At least 3 animals of each genotype were analyzed.
Data exclusions	A few animals were excluded from survival curves due to fight wounds, malocclusion, dystocia, or genotype-independent hypotrophy.
Replication	All findings were confirmed by at least two independent experiments including at least 3 animals of each genotypes or at least three independent in vitro assays.
Randomization	For in vivo experiments, animals were allocated to different experimental groups based on gender and age to equal distribution.
Blinding	In general, all animal experiments were under a blinded condition because mice were identified only by mice ID but not genotype during data collection.

Reporting for specific materials, systems and methods

We require information from authors about some types of materials, experimental systems and methods used in many studies. Here, indicate whether each material, system or method listed is relevant to your study. If you are not sure if a list item applies to your research, read the appropriate section before selecting a response.

Materials & experimental systems

n/a	Involved in the study	
☐	☒	Antibodies
☐	☒	Eukaryotic cell lines
☒	☐	Palaeontology
☐	☒	Animals and other organisms
☒	☐	Human research participants
☒	☐	Clinical data

Methods

n/a	Involved in the study	
☒	☐	ChIP-seq
☐	☒	Flow cytometry
☒	☐	MRI-based neuroimaging

Antibodies

Antibodies used	Primary antibodies used for immunostaining are: anti-CD3ε (Dianova, HH3E, DIA-303), anti-Keratin14 (Covance, PRB-155P), anti-F4/80 (BIO-RAD, Cl:A3-1, MCA497GA), and anti-myeloperoxidase (MPO) (ThermoFisher Scientific, RB-373-A1). primary antibodies used for in vitro cell death neutralization assay are: anti-TNFα (XT3.11) and a rat IgG1 isotype control (HRPN) from Bio X cell. The followed antibodies are used for western blot analysis: anti-Sharpin, anti-HOIL-1L, and anti-HOIP, home-maded; anti-α-tubulin (DM1A, CLT9002) from Cadarlane Laboratories; anti-β-actin (AC-74, A5316) from Sigma. All the followed antibodies were used for Flow cytometry analysis: anti- anti-CD25 (PC61, 102006), anti-IFNγ (XMG1.2, 505806), anti-CD103 (2E7, 121420), anti-GITR (YGITR765, 120205), anti-CD44 (IM7, 103008), anti-IL-4 (11B11, 504104), anti-CD4 (GK1.5, 100434), anti-CD25 (PC61, 102029), anti-IL-5 (TRFK5, 504303), anti-CD62L (MEL-14, 104412), anti-CD69 (H1.2F3, 104513), anti-Gr1 (RB6-8C5, 108411), anti-CD103 (2E7, 121403), anti-IL-17A (TC11-18H10.1, 506916), anti-GITR (DTA-1, 126311), anti-CD304 (Nrp-1) (3E12, 145209), anti-CD3ε (145-2C11, 100334), anti-CD19 (6D5, 115519), anti-CD152 (CTLA4) (UC10-4B9, 106313), anti-B220 (RA3-6B2, 103224), streptavidin-PerCP (405213), anti-KLRG1 (2F1/KLRG1, 138409), anti-CD11b (M1/70, 101215), anti-Helios (22F6, 137216), anti-

CD154 (CD40L) (MR1, 106505), anti-CD120a (TNFRI) (55R-286, 113003), anti-CD120b (TNFRII) (TR75-89, 113405), anti-CD262 (DR5, TRAIL-R2) (MD5-1, 119905), and anti-CD3ε (145-2C11, 100333), all from BioLegend; anti-CD3ε (145-2C11, 553062), anti-CD11b (M1/70, 553310), anti-B220 (RA3-6B2, 11-0452-82), anti-CD5 (53-7.3, 553022), anti-CD25 (PC61, 552880), anti-CD8α (53-6.7, 552877), anti-CD19 (1D3, 562701), streptavidin-APC-Cy7 (554063), anti-CD69 (H1.2F3, 553237), anti-CD95 (Fas) (Jo2, 554258), and anti-SiglecF (E50-2440, 552126), all from BD Biosciences; anti-TCRβ (H57-597, 11-5961-82), anti-CD25 (PC61.5, 12-0251-82), anti-Ki-67 (SolA15, 12-5698-80), anti-IFNγ (XMG1.2, 12-7311-81), anti-CD4 (RM4-5, 17-0042-82), anti-B220 (RA3-6B2, 17-0452-83), anti-IL-13 (eBio13A, 50-7133-80), anti-γδTCR (eBioGL3, 13-5711-81), anti-FoxP3 (FJK-16s, 17-5773-82), streptavidin-APC (17-4317-82), anti-NK1.1 (PK136, 17-5941-81), anti-CD45RB (C363.16A, 12-0455-82), anti-Foxp3 (FJK-16s, 11-5773-82), anti-CD19 (eBio1D3, 11-0193-82), anti-TCRβ (H57-597, 12-5961-82), anti-IL-2 (JES6-5H4, 12-7021-81), streptavidin-PE (12-4317-87), anti-CD62L (MEL-14, 13-0621-82), anti-NK1.1 (PK136, 13-5941-82), anti-TNFα (MP6-XT22, 17-7321-81), anti-IL-10 (JES5-16E3, 17-7101-81), and anti-IFNγ (XMG1.2, 17-7311-81), all from eBioscience; and anti-CD127 (A7R34, 50-1271-U025), from TONBO Biosciences.

Validation

All antibodies are applicable to the experiments using mice. Antibodies against Sharpin, HOIL-1L, HOIP, α-Tubulin, and β-actin are used for western blot experiment. anti-TNFα (XT3.11) and a rat IgG1 isotype control (HRPN) are used for in vitro neutralization assay, and the other antibodies are applicable to Flow Cytometry analysis.

Eukaryotic cell lines

Policy information about cell lines

Cell line source(s)

B3Z T cell hybridoma and H2Kb-expressing L cells (K89) are described in paper from Dr. Nilabh Shastri (University of California). HT-2 cells were obtained from Eric Huseby (University of Massachusetts Medical School). Murine embryonic Fibroblasts used in this study were previously established in our laboratory.

Authentication

None of the cell lines used were not authenticated.

Mycoplasma contamination

None of the cell lines used were not tested for mycoplasma.

Commonly misidentified lines
(See ICLAC register)

No commonly misidentified cell line was used.

Animals and other organisms

Policy information about studies involving animals; ARRIVE guidelines recommended for reporting animal research

Laboratory animals

Mice were bred and housed in a full barrier, specific pathogen free (SPF) animal facility at Kyoto University and RIKEN Kobe Branch. Information regarding animals is available in the Methods section and Figure legends. In general, age-matched male and female adult mice were used and mixed for analyses of experiments except for analyses of disease course.

Wild animals

This study did not involve the wild animals.

Field-collected samples

This study did not involve samples collected from the field.

Ethics oversight

All animal studies were approved by Animal Research Committee, Graduate School of Medicine, Kyoto University.

Note that full information on the approval of the study protocol must also be provided in the manuscript.

Flow Cytometry

Plots

Confirm that:

☒ The axis labels state the marker and fluorochrome used (e.g. CD4-FITC).

☒ The axis scales are clearly visible. Include numbers along axes only for bottom left plot of group (a 'group' is an analysis of identical markers).

☒ All plots are contour plots with outliers or pseudocolor plots.

☒ A numerical value for number of cells or percentage (with statistics) is provided.

Methodology

Sample preparation

Single cell suspenstions of thymocytes, splenocytes, and lymph node cells were prepared from dissected tissues by grinding with 5ml syringe piston (Terumo) on a 70 µm cell strainer (Falcon). Cells infiltrating the liver were isolated by mechanical dissociation using a 5ml syringe piston (Terumo) on a 100 µm cell strainer (Falcon), followed by purification on a 33% Percoll solution (Sigma) (800g, 30 min at room temp). Skin-resident cells were isolated separately from the dermis or epidermis. After shaving, the skin was immersed in 0.5 g/ml Dispase II (Wako) diluted in PBS and left at 4°C overnight. The epidermal layer was then peeled from the dermis. Epidermal tissues were treated at 37°C for 10 min with 0.05% DNase I (Roche) containing trypsin solution. Dermal tissues were incubated with 0.13 units/ml Liberase TM (Roche) and 100 µg/ml DNase I (Roche) diluted in plain RPMI and then rotated at 37°C for 40 min. Before staining for the purifications of naive T cells or Tregs by FACSAria, isolated single cell suspensions were mixed with antibody-conjugated MACS beads to remove CD8+ and CD19+ cells. Red blood cell lysis was

performed on samples from spleen and liver. The cell suspension obtained from each tissue was filtered through 100-micron nylon filter mesh to remove cell aggregates and clumps.

Instrument	Skin, Spleen, peripheral lymph nodes, liver, and intestine FACS analysis data was collected on BD Canto II analyzer (Bectin Dickinson). Cell purification for T cell-induced colitis experiments and cloning of crispr-based knockout cell lines was accomplished in a FACS Aria III (Bectin Dickinson).
Software	Data were collected with FACS Diva software (Becton Dickinson), and the established files were subsequently analyzed using FlowJo software (Tomy Digital Biology).
Cell population abundance	Post-sorted subsets of spleen and peripheral lymph node immune cells were immediately checked that the population of the cells were greater than 98% pure.
Gating strategy	Dead cells were excluded based on FSC-A and SSC-A characteristics and using LIVE/DEAD Fixable Violet Dead Cell Stain kit (ThermoFisher Scientific). Cell populations were gated as follows: Treg (CD3e+CD4+CD25+YFP+ [or Foxp3+]), conventional CD4T (CD3e+CD4+CD25-YFP- [or Foxp3-]), intestinal T cells (CD3+CD4+), eosinophils (CD11b+SiglecF+), neutrophils (CD11b+Gr1hi), granulocytes (CD11b+Gr1+), Keratinocytes (CD45-Lineage- [CD11b-CD3e-B220-Ly6G/C-TER119-]), skin whole T cells (CD45 +Thy1.2+), epidermal TCRb T cell (CD3+TCRb+TCRgd-), epidermal TCRgd T cells (CD3ehiTCRb-TCRgd+), resident memory T cells (CD3e+TCRb+CD69+CD103+CD62Llo), apoptotic keratinocytes (CD45-Lineage- [CD11b-CD3e-B220-Ly6G/C-TER119-] PhiPhiLux-G1D2+), Naive T cells (CD3e+CD4+CD25-CD45RB+).

☐ Tick this box to confirm that a figure exemplifying the gating strategy is provided in the Supplementary Information.
